# The complete mitochondrial genome of Yihe bream, *Megalobrama amblycephala* Yih

**DOI:** 10.1080/23802359.2017.1365652

**Published:** 2017-08-22

**Authors:** Guojun Guo, Shaokui Yi, Weimin Wang

**Affiliations:** a College of Animal Science and Technology, Henan University of Animal Husbandry and Economy, Zhengzhou, China;; b College of Fisheries, Huazhong Agricultural University, Wuhan, China

**Keywords:** Mitochondrial genome, *Megalobrama amblycephala*, Yihe bream, phylogenetic tree

## Abstract

The evolutionary status and phylogenetic relationships of *Megalobrama* species remain unclear, despite the efforts. The genetic information of Yihe bream, a new member in *Megalobrama*, is quite limited. The complete mitochondrial genome of Yihe bream was urgent to reveal the genetic relationships and evolutionary status. In this study, we sequenced the mitochondrial genome of Yihe bream. The genome is 16,624bp in length and structurally identical to the other *Megalobrama* species. The phylogenetic tree showed that Yihe bream clustered with blunt snout bream, indicated the closer genetic relationships between these two species. The genome of Yihe bream would be useful for the future researches of population genetics, evolution and DNA barcoding of *Megalobrama* species.

Yihe bream (*Megalobrama amblycephala* Yih), which is only distributed in a tributary of Yellow River named Yi River, were described in Chinese history records nearly 1500 years ago. Since 1920s, the fishery resources of Yi River have been investigated, and the distribution area of Yihe bream was firstly defined. The morphology and karyotype of Yihe bream have been proposed in the previous studies (Qu et al. 2013; Zhang et al. 2014), and Yihe bream is considered as a subspecies of blunt snout bream. In this study, we report the complete mitochondrial genome sequence of *M. amblycephala* Yih to provide useful genetic information for the future study in conversation biology and phylogenetic analysis.

The sample of *M. amblycephala* Yih was collected from Luhun Reservoir (N34°10′25.50″, E112°09′43.81″) in the middle reaches of Yi River. After morphological identification, the specimens were kept in the laboratory of Haid institute at −80 °C under the accession number YHF201706005. DNA was extracted from caudal fin tissue using a modified amonium acetate precipitation protocol (Nishiguchi et al. 2002). Fourteen pairs of PCR primers were designed to amplify the whole mitochondrial genome sequence based on the conserved sequences from other close species. The mitochondrial genome of *M. amblycephala* Yih (GenBank accession number MF522177) was a circular molecule of 16,623 nucleotides. The overall nucleotide composition was 31.3% A, 24.7% T, 27.9% C and 16.2% G, with an AT content of 56.0%. This mitochondrial genome had a mostly conserved structural organization when compared with that of other teleost fish, including 13 protein-coding genes (PCGs), 22 transfer RNA genes, 2 ribosomal RNA (12 S rRNA and 16 S rRNA) genes, and 2 main non-coding regions (the control region and the origin of the light strand replication). Protein-encoding genes include ATP-synthase subunits 6, 8, and 9 (atp6, atp8, and atp9), cytochrome oxidase subunits (cox1, cox2, and cox3), cytochrome b (cytb), and NADH dehydrogenase subunits (nad1, nad2, nad3, nad4, nad4L, nad5, and nad6) whose products involved in mitochondrial electron transfer, oxidative phosphorylation and mitochondrial protein synthesis. The gene order of *M. amblycephala* Yih was identical with that of the known representatives of the mitochondrial genomes of the species in genus *Megalobrama* (Lai et al. 2014). Most of the mitochondrial genes encoded on heavy strand (H-strand) except for ND6 and eight tRNA genes, which are encoded on light strand (L-strand).

Phylogenetic analysis based on the concatenated nucleotide sequence of 13 PCGs confirms *M. amblycephala* Yih as a member of the genus *Megalobrama*. Moreover, *M. amblycephala* Yih is clustered with *M. amblycephala*, *M. pellegrini* and *M. terminalis* according to our phylogenetic analysis (Figure 1), with consistent taxonomic status according to phylogenetic analysis in the previous study (Lai et al. 2014).

**Figure 1.**
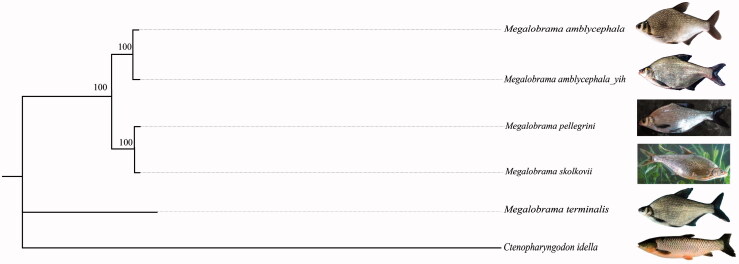
Phylogenetic analysis of *M. amblycephala* Yih and mitochondrial genome of the related species in genus *Megalobrama*. The NJ tree is based on the concatenated sequences of 13 PCGs. *Ctenopharyngodon idella* was used as outgroup. Sequence alignment and phylogenetic analyses were conducted using MEGA7 (Kumar et al. 2016).
